# Managing residents in difficulty within CBME residency educational systems: a scoping review

**DOI:** 10.1186/s12909-020-02150-0

**Published:** 2020-07-23

**Authors:** Jonathan Pirie, Lisa St. Amant, Susan Glover Takahashi

**Affiliations:** 1 Department of Pediatrics, Faculty of Medicine, University of Toronto, Toronto, Canada; 2 Paediatric Emergency Medicine, The Hospital for Sick Children, Toronto, Canada; 3 Postgraduate Medical Education, Faculty of Medicine, University of Toronto, Toronto, Canada; 4 Department of Family and Community Medicine, Faculty of Medicine, Integrated Senior Scholar – Centre for Faculty Development and Postgraduate Medical Education, University of Toronto, Toronto, Canada

**Keywords:** Remediation, Residents in difficulty, Competency-based medical education

## Abstract

**Background:**

Best practices in managing residents in difficulty (RID) in the era of competency-based medical education (CBME) are not well described. This scoping review aimed to inventory the current literature and identify major themes in the articles that address or employ CBME as part of the identification and remediation of residents in difficulty.

**Methods:**

Articles published between 2011 to 2017 were included if they were about postgraduate medical education, RID, and offered information to inform the structure and/or processes of CBME. All three reviewers performed a primary screening, followed by a secondary screening of abstracts of the chosen articles, and then a final comprehensive sub-analysis of the 11 articles identified as using a CBME framework.

**Results:**

Of 165 articles initially identified, 92 qualified for secondary screening; the 63 remaining articles underwent full-text abstracting. Ten themes were identified from the content analysis with “identification of RID” (41%) and “defining and classifying deficiencies” (30%) being the most frequent. In the CBME article sub-analysis, the most frequent themes were: need to identify RID (64%), improving assessment tools (45%), and roles and responsibilities of players involved in remediation (27%). Almost half of the CBME articles were published in 2016–2017.

**Conclusions:**

Although CBME programs have been implemented for many years, articles have only recently begun specifically addressing RID within a competency framework. Much work is needed to describe the sequenced progression, tailored learning experiences, and competency-focused instruction. Finally, future research should focus on the outcomes of remediation in CBME programs.

## Background

Best practices for managing residents in difficulty are neither well defined nor consistently applied. The term “residents in difficulty” generally refers to graduate medical trainees who have demonstrated a significant or sustained pattern of underperformance compared to expectations. As postgraduate medical education training programs transition to competency-based medical education (CBME), the need for definitions and approaches may increase.

In a recent article about CBME, Van Melle [1] et al. identified five key features of CBME educational designs: 1) an outcomes-based competency framework; 2) using a sequenced progression of competence; 3) including tailored learning experiences for the achievement of competencies; 4) tailoring teaching to competencies that, for residents, include multiple workplace observations followed by feedback and coaching; and 5) taking a purposeful and programmatic approach to assessment. We will explore whether the literature about residents in difficulty considers these features of CBME.

Several articles attempted to quantify the prevalence, characteristics, areas of weakness, and outcomes of residents on remediation or probation. Zbieranowski [2] and Guerrasio [3] each looked at a 10-year period at one university, ending in 2009 and 2012 respectively. In both articles, competency-based models were not in effect during the eras studied. Dupras [4] reported on a survey across Internal Medicine (IM) programs that sought to define prevalence, problems, and remediation in the era of core competencies, which preceded the roll-out of CBME residency training programs.

Very little literature exists on residents in difficulty within the context of CBME programs. Past reviews have looked at prevalence, which competencies residents underperform in, and what interventions are used.

This scoping review takes stock of what is known about residents in difficulty within the CBME context and connects it to the available literature on best practices for residents in difficulty. In North America, where the vast majority of the identified studies were published, CBME was first launched in the United States by the Accreditation Council for Graduate Medical Education (ACGME) in the 6 Core Competencies in 2001 and more specifically developed through the Milestones Project since 2013 [5]. In Canada, the College of Family Physicians of Canada (CFPC) launched their Triple C curriculum in 2011 [6]; and the Royal College of Physicians and Surgeons (RCPSC) adopted the CanMEDS framework in 1996, updating it in 2005 and 2015 [7], before developing a more structured CBME curriculum, Competence by Design, implemented gradually beginning in 2017 [8]. Specifically, we considered papers about residents in difficulty in a pre- and post-CBME context and considered what has been written on such topics as identifying a resident in difficulty, implementing remedial education plans, monitoring resident progress during the remedial period, and determining successful resident outcomes following the remedial period.

The purpose of this paper is to provide a review of the current literature on residents in difficulty, spanning the era of the introduction of CBME to post-graduate training programs. The specific objectives of this scoping review were to

- inventory current literature
- identify major themes
- report on the frequency and types of articles that address CBME as part of the identification and remediation of residents in difficulty.

## Methods

A scoping review methodology, as outlined by Arksey and O’Malley [9] and Levac et al. [10] (2010), was used to provide a comprehensive overview of the current literature on residents in difficulty, spanning the era of the introduction of CBME to post-graduate training programs.

The main stages in our methodology consisted of 1) identifying the research question; 2) developing a search strategy for capturing literature relevant to the research question; 3) screening the literature for inclusion; 4) analyzing the results using a grounded theory approach; and 5) collating, summarizing, and reporting of the results.

We employed multiple quality assurance processes, including calibration of reviewers during the screening, abstracting, and coding stages. The three reviewers performed calibration exercises to achieve clarification and consensus around the screening and abstracting criteria and outcomes (e.g. What constitutes as inclusion? What is meant by the abstracting criteria? What information is being captured / excluded?). For example, during the article title and abstracting stage, the three reviewers were assigned six articles each to screen for inclusion (‘yes’, ‘no’ or ‘unsure’), with each article being reviewed by two separate reviewers. Once screened, the reviewers met to discuss the results, discrepancies and questions that arose. This process was repeated until consensus was reached and the reviewers were confident to begin screening articles independently. The same process was applied for article abstracting. Throughout the screening and abstracting processes, reviewers were encouraged to flag articles as “unsure” or “in need of discussion” for discussion as a group. During qualitative analysis, a similar calibration exercise was used to establish the article coding framework for identifying themes across articles and findings of interest / impact.

The key research question was: *What does the current literature say about the remediation*^1^*of graduate medical trainees using competency-based educational approaches?*

### Inclusion criteria

We first established our article inclusion criteria to narrow down the body of literature to articles solely on our topic of interest. The three criteria are listed and described in Table 1.

**Table 1:** List and description of review inclusion criteria

	Inclusion criteria	Description
1	Must be about postgraduate medical education	Includes any specialty / program area
2	Must be about residents in difficulty	Includes discussion on remediation and Board of Examiners (BOE)^a^ cases (e.g. definitions and descriptions to guide the identification of residents in difficulty; discussions on remedial support or BOE remediation; discussions of documentation needed to verify resident needs and program processes for BOE)
3	Must offer information to inform structure and/or processes of competence (including competency-based education)	Structure includes guidelines, program design, promotion and progress; Processes include features of competence (e.g. the CanMEDS Roles that are involved)

^a^The Board of Examiners for postgraduate medicine (BOE-PG) is a University-level committee of faculty and residents appointed to adjudicate on the educational programming and/or future registration status for residents identified by residency programs due to a pattern of underperformance, failing performance or serious behaviours. Following a structured process of reviewing of the situation and opportunity for resident consultation and response, the BOE determine the best course(s) of action such as remediation, remediation with probation, probation or suspension and dismissal

### Literature search

We searched the electronic databases of ERIC, MEDLINE, and EMBASE, with the aid of the Department of Family and Community Medicine (DFCM) librarian, using a search framework from a previous literature review conducted by two members of the research team (SGT, LSA) (See Supplement A for sample search strategy). Sample keywords used to search various article fields including titles, abstracts, subject heading and author keywords included: “physician,” “trainee,” “intern,” “residency”, “house officer”, “remediation,” “academic difficulty” and “residents in difficulty” (along with all relevant wildcards, plural forms, and other relevant terms). The subject headings of “education, medical, graduate/” and “remedial teaching” were also used. By combining search terms, articles were only included if they had discussed both residency education and academic difficulty / remediation, accounting for a narrower scope (See Supplement B for Sample Search Strategy).

The last major literature review on remediation, conducted by Cleland et al. [11], looked at articles from 1984 to 2012, inclusive. Thus, we chose to focus our scoping review on the most recent body of literature, published from 2011 to 2017, inclusive. Another reason for that focus was that residency education has only very recently moved purposefully toward implementing CBME systems.

### Primary screen

Of the 165 articles identified from the literature search, 131 were included in the primary screen (34 were duplicates and therefore excluded). Three reviewers (LSA, MR,^2^ and SGT) were calibrated on the primary screening of 10 of the 131 article titles and abstracts to ensure screening consistency and to refine the screening criteria as necessary. The remaining article titles and abstracts were divided among the members of the research team and screened independently.

### Secondary screen and data extracting

The team brainstormed on the type of data to collect that would be relevant to our topic, and to the aims of our local Best Practices in Evaluation and Assessment (BPEA) Working Group^3^ at large. We constructed an online form to collect data of interest from included full-text articles. (See Supplement C for complete list of data extraction criteria, their descriptions, and examples from the reviewed literature).

A second calibration exercise was performed with three reviewers (JP, LSA, and MR) on the screening and abstracting of five of the 92 full-text articles included from the primary screen. This helped us to ensure consistency among reviewers in the screening of full-text articles, refine the data abstracting tool, and form a common understanding of the extraction criteria. We improved and expanded upon the data extraction criteria as a result.

The remaining full-text articles were divided among the team (JP, LSA, and MR) to be screened and abstracted independently.

### Descriptive and qualitative summary of results

Data collected from eligible articles were summarized descriptively (frequencies, crosstabulations), using IBM SPSS Statistics Software v24. Qualitative data were collected about each article’s purpose or objective(s); the main findings related to the structures to support competence that were discussed; and any other findings related to residents in difficulty, remediation, and/or BOE. All qualitative data were analyzed with NVivo v11 using summative content analysis methodology, as described by Hsieh and Shannon [12], to identify major themes.

A secondary, more comprehensive analysis was done of the 11 articles that discussed or used a CBME orientation or framework.

### Risk of Bias

In such research, there is the potential for reviewer bias during the primary and secondary screens, as well as the qualitative analysis. To mitigate reviewer bias, calibration exercises were performed at each stage until all reviewers achieved consensus; and the themes and abstracting criteria were discussed, iteratively reviewed and refined throughout. There is also inherent bias due to the criteria for inclusion selected by the authors, which might have excluded other relevant studies from this review. Lastly, there is also the risk of bias in the gathered articles due to the interests of publishers and researchers, alike, thereby possibly limiting completed understanding and/or coverage of these topics.

## Results

### Abstracting results

**Fig. 1:**
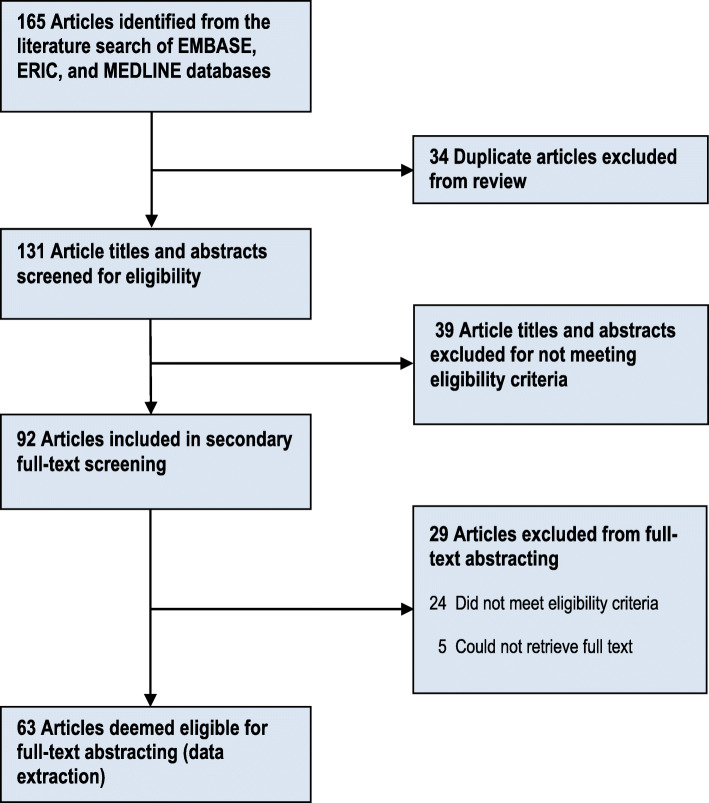
Selection process used in a review of the literature published from 2011 to 2017

Figure 1 shows the literature review screening process and results. Of the 165 articles identified from our literature search, 92 article titles and abstracts passed the primary screen and went on to secondary screening. A total of 63 full-text articles were included from the secondary screen and underwent data extraction and analysis.

### Descriptive overview of included articles (*n* = 63)

This section outlines some key results based on frequency of occurrence. Note that frequency does not infer importance per se, but rather reflects the topics and contexts of greatest interest to researchers and publishers.

**Fig. 2:**
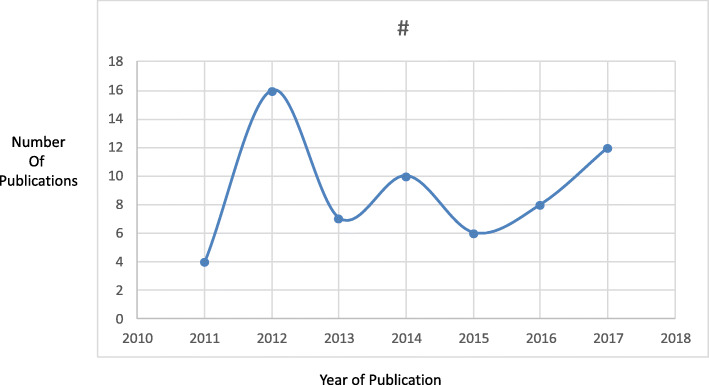
Frequency of articles by year of publication

Interestingly, the year 2012 saw the most articles published on residents in difficulty, remediation, and/or BOE (*n* = 16) (see Fig. 2). Most articles reviewed were primary research studies (*n* = 36, 57%) — e.g. correlation, observation, and prediction studies — with only 5 review articles captured (8%). The majority of articles were published in the United States (*n* = 45, 71%).

The specialties most often discussed were Emergency Medicine (*n* = 11, 17%), Surgery (*n* = 11, 17%), Family Medicine (*n* = 6, 10%), and Internal Medicine (*n* = 6, 10%); a quarter of articles (*n* = 15, 24%) were not associated with any particular specialty.

Medical Expert (*n* = 33, 52%), Professionalism (*n* = 24, 38%), and Communicator (*n* = 11, 17%) were the competencies most often discussed in association with residents in difficulty, remediation, and/or BOE.

“Guidelines for program” was the structure most frequently used to support resident competence (*n* = 45, 71%). Other commonly discussed structures were: “Design of residency educational program” (*n* = 21, 33%) and “Design of individual resident educational plan/program” (*n* = 19, 30%). Many articles that discussed “guidelines for program” were primary research studies (*n* = 24, 38%). “Promotion of resident systems” was **rarely** discussed (*n* = 7, 11%); it was defined as the system(s) in place for promoting residents from one stage of training or PG year to the next (e.g. How are promotion decisions made? What data points inform the decision to promote residents? How is competence assessed?).

### Qualitative summary

Ten themes were identified from the content analysis of qualitative data collected on all 63 reviewed articles (Table 2). “Identification of deficiencies” (*n* = 33, 52%) and the “importance of defining and classifying resident problems or deficiencies as a first step to remediating them” (*n* = 19, 30%) were the two most common themes identified. Additionally, the subgroup of papers that were explicit about remediation within a CBE context were analysed to see whether they differed from the 10 themes identified and if the emphasis within the subgroup was consistent or not.

**Table 2:** List and Description of Themes Identified from Qualitative Content Analysis

Primary Themes		Secondary Themes	Description	Examples	Frequency			
					All Articles (*N* = 63)		CBME Articles (*N* = 11)	
					#	%	#	%
**1.**	Identification of residents in difficulty [3–21]	**A.** Assessment	Includes articles discussing the association between assessment tool type, accuracy or frequency, and the identification of residents in difficulty / in need of remediation	The utility of Standardized Direct Observation Tools (SDOT) and OSCEs to identify deficiencies in clinical performance (Medical Expert)	26	41%	7	64%
		**B.** Faculty Development	Includes articles discussing how faculty development / training could help improve faculty’s ability to diagnose resident difficulties / deficiencies	Through improving the knowledge and skills of faculty around proper assessment and feedback methods; teaching faculty how to define and classify deficiencies so they can be more readily identified in practice	8	13%	1	9%
		**C.** Other	Other topics discussed in relation to the identification of resident deficiencies	Factors predictive of resident deficiencies / their need for remediation in the future (e.g. age of application to residency, having transferred from another institution) [18]; identification of deficiencies by creating and having a better understanding of the taxonomy of the “problem resident” [19, 20]	3	5%	0	0%
**2.**	Defining and classifying resident deficiencies		Discusses the importance of creating a framework for defining and classifying resident problems in an effort to design appropriate programs tailored to the issue(s) at hand. Addresses the questions of “What is the problem or deficiency?”; “What are its causes?”; “What signals a deficiency in this competency domain?”; etc.	Defining the problem through subjective and objective measures	19	30%	3	27%
				Classifying problems, e.g. by rating their severity, determining whether the problem is inherent or contextual, what the contributing factors are				
**3.**	Improving assessment tools and/or methods for tracking the progress of residents undergoing remediation		Improving the accuracy and/or frequency of assessment to better track residents’ progress throughout their remediation	Advocating for more frequent formal and informal evaluations and feedback	10	16%	5	45%
				Often found to improve resident learning outcomes				
**4.**	Individualizing or tailoring of the remediation plan / program	**A.** To the resident	Many articles discuss the importance of tailoring remediation plans to the resident	Customizing the approach and structure and the resources needed for the remediation plan for each resident, with consideration given to the problem type and severity, and the resident’s characteristics (such as their learning style, personality, and level of insight).	16	25%	3	27%
		**B.** To the specialty	Different specialties may require special consideration when developing a remediation plan, due to the unique nature of their training program and/or special clinical/training environment(s)	Emergency Medicine interns are found to have a significantly higher chance of under-performing than those in other disciplines, thought to be possibly related to differences in assessment practices and/or training environments	3	5%	2	18%
**5.**	Defining terms relating to remediation		Demonstrates the importance of having institutional-level consistency in the definitions of terms relating to remediation. This can help Program Directors (PDs), their faculty, and residents to better understand the expectations for training and the repercussions of not meeting them	Need to better differentiate and understand the difference between “need for improvement,” “need for remediation” (formal vs. informal), and “need for probation” (notice of potential for dismissal), e.g. CanMEDS, ACGME Roles	8	13%	2	18%
**6.**	Demands placed on faculty by remediation		Any strain on faculty as a result of their participation in the remediation of residents, including their time and effort spent and the complexity of their role(s)	Most refer to how time-consuming it is for faculty to participate in remediation programs and the great effort usually required	9	14%	2	18%
**7.**	Hidden curriculum		Discusses the “hidden curriculum” in terms of attending role modelling, which can either positively or negatively impact residents	Faculty and residents need to be held to the same standards of professionalism, e.g. studies find that residents are given passes on their behaviour relative to the learner’s level of training	2	3%	0	0%
**8.**	Associations with past performance		Correlations observed between past performance (e.g. in medical school) and performance during residency	Performance in medical school may be predictive of performance in residency	7	11%	1	9%
**9.**	Pilot testing of plan / program		Discusses the pilot testing of a novel remediation plan or program, including program description and program effectiveness and/or outcomes	Looks at the effects of a 4-month training program implemented in an Emergency Medicine residency program to improve residents’ American Board of Emergency Medicine exam scores	3	5%	0	0%
**10.**	Roles and responsibilities of players involved in remediation		Understanding and identifying the many “players” involved in remediating residents, in identifying those in difficulty, and defining their roles	Identifying the key individuals to be involved in the remediation process and specifying their roles for varying severities of resident problems, e.G. *minor* problems can be managed by the ward or department, whereas more serious problems might merit a formal investigation	7	11%	3	27%
**11.**	Other		Topic areas that were less prominent in the literature reviewed	Critique of literature on remediation (review); PD survey of incidence / prevalence of resident problems and possible predictors; plan for improved self-reflection integrated into remediation programs; general description of remedial process; and benchmark scale for residency training	13	21%	3	27%

The top two themes arising from our qualitative analysis are explored in greater detail below.

### Identification of residents in difficulty

Early assessment and identification of resident problems is considered essential to the success of remediation efforts in terms of resident learning and behavioural outcomes. Early identification has the following impacts: minimizes the use of resources (deficiencies increase in severity the longer they persist in training, thus requiring more time and effort to remediate); reduces negative impacts to patient safety and quality of care (identifying and remediating resident problems earlier means less exposure of underperforming residents to patients); decreases negative effects on the functioning of health professional teams and the system at large (health professional teams are negatively affected by the actions of underperforming residents).

Many types of assessments were discussed that could be used to identify resident deficiencies, such as:

- In-training specialty exams, especially if occurring early in the learning experience or residency year [13]
- Innovative in-training assessment tools for providing formative feedback, such as the Clinical Skills Verification program described by Dalack and Jibson [14, 15]
- Objective Standardized Clinical Examinations (OSCEs) to uncover knowledge gaps in trainees [15]
- Standardized Direct Observation Tools (SDOT) [16]
- Simulation laboratories [16]
- Simulated oral board cases [16]
- Post-curriculum multiple choice examinations, administered yearly [17]
- TriMetrix, a tool used to measure the behaviours and motivations of residents [18]
- Administering pre- and post-lecture questions to allow early identification of gaps in medical knowledge [19]
- General faculty evaluations [20]

Besides early targeted assessments, almost a quarter of the articles deemed faculty development important for identifying residents in difficulty (21%, *n* = 4/19) [21–24].

#### Defining and Classifying Deficiencies

Separate from the identification of residents in difficulty is the need to have a literature-informed standardized classification system for categorizing and defining the many different kinds of resident deficiencies.

Defining and classifying resident problems includes rating the level of seriousness of the deficiency [25] and exploring potential causes [26] (e.g. mental health issues, cognitive disabilities).

Having standardized definitions and a means of classifying deficiencies is necessary for developing more uniform approaches to remediation that are targeted to improving this issue [27]. Such means also allow Program Directors and faculty assessors to have a mental model or framework for identifying these deficiencies in practice.

Repeatedly, articles discussed the importance of defining and classifying as a necessary first step to understanding the best course of action to take in improving a resident’s performance to meet the standards of the program, whether it is through informal coaching, mentoring and monitoring of progress, more formal remediation, or probation if the issue is non-remediable.

Some of the literature we reviewed offered categorizations and definitions of commonly occurring resident problems, or referenced other articles that did, such as surgical clinical performance [28], professional behaviour, and competence and collaboration [25, 29–31]. Information on these articles can be found in Supplement D.

### CBME article sub-analysis (11 articles)

Competency-based medical education was explicitly discussed infrequently. Eleven (17%) articles discussed a competency-based educational orientation or framework. Half of these papers were from the last 2 years (2016–2017), reflecting the increasing global interest in CBME.

### Themes

The most common themes were similar in the CBME-related articles: “Identification of residents in difficulty” (64%) and “Improving assessment tools for tracking progress of residents undergoing remediation” (45%). The theme “Roles and responsibilities of players involved in remediation” was also cited frequently (27%) in CBME-related articles, often describing the structure and role of clinical competency committees. The frequency of themes did not differ significantly from non-CBME–related articles, although the numbers are small, making comparisons difficult.

Below is a summary of some of the articles representing these themes (Theme #):

1. Identifying residents in difficulty (1. A. Assessment):

**R. Domen et al. Resident Remediation, Probation, and Dismissal. 2014.**

Describes a new approach for pathology training programs. They discuss the identification of resident performance problems using close observation by multiple observers, using multiple evaluation tools with 360-degree evaluation, at multiple points during the training period. They describe an eight-step remediation development plan and see the burden of responsibility lying between PDs and CCC [24].

**B. Kinnear et al. Critical Deficiency Ratings in Milestone Assessment: A Review and Case Study. 2017.**

Examines the proportion of ACGME milestone sets that include critical deficiencies (CD) ratings and describes one internal medicine residency program’s experiences using CD ratings in assessment. Identification of CDs may help programs develop remediation and improvement plans [32].

**K. Walburton et al. Comprehensive Assessment of Struggling Learners Referred to a Graduate Medical Education Remediation Program. 2017.**

Describes the development of and composition of an Early Intervention Remediation Committee (EIRC), a process for identifying struggling learners (RID), the categorizing of primary deficits, the assessment of learners, and the outcomes. Over 2 years, 4% of learners required remediation plans and all were successfully completed. Time commitment averaged 45 h per learner [33].

2. Assessment and improving assessment tools and methods for tracking progress (3.)

**B. Gas et al. Objective Assessment of General Surgery Residents Followed by Remediation. 2016.**

Describes the development of an objective assessment of general surgery residents using a five-station skills assessment event called “X-Games.” Those residents who failed stations (89%) were “remediated” with take-home and deliberate practice, scoring rubrics, and video re-assessment. Of the residents who attempted remediation (58%), all achieved a score greater than or equal to 4/5 [34].

**L. McMurray et al. The Nightmares Course: A Longitudinal, Multidisciplinary, Simulation-Based Curriculum to Train and Assess Resident Competence in Resuscitation. 2017.**

Describes the use of a formative simulation-based resuscitation “Nightmares” course: a novel, competency-based, transitional curriculum and assessment program in resuscitation medicine with competency-based thresholds using entrustable decisions. This was followed by a summative OSCE in which 35% of residents did not meet the competency threshold and subsequently participated in remediation sessions [35].

3. Roles and responsibilities of players involved in remediation (10.)

**E. Ketteler et al. Competency Champions in the Clinical Competency Committee: A Successful Strategy to Implement Milestone Evaluations and Competency Coaching. 2014.**

Describes the development of a CCC for surgical residents. The role of the CCC was to identify RID, the players/roles of the members, and the frequency of evaluation, using the ACGME competencies. The selection of “competency champions” is described, along with the process to identify RID who require remediation and more formal performance evaluation [36].

**J. Smith et al. Defining Uniform Processes for Remediation, Probation and Termination in Residency Training. 2017.**

An attempt to establish standardized definitions of levels of RID, management processes, documentation expectations, and appropriate notifications. The authors then develop a “remediation schema for residents at risk of not meeting educational milestones during their training,” including the roles of the PD, the designated institutional offices (DIO), and the GME offices [37].

**K. Walburton et al. Comprehensive Assessment of Struggling Learners Referred to a Graduate Medical Education Remediation Program. 2017 (see 1. above)** [33].

4. Other areas included (11.)

**D. Dupras et al. “Problem Residents”: Prevalence, Problems and Remediation in the Era of Core Competencies. 2012.**

The purpose here was to assess the experiences of internal medicine PDs, including the incidence and prevalence of resident problems in programs using ACGME competencies. The study concludes that identification of RID is difficult prior to residency, that residents have difficulties in multiple competencies, and have the most trouble with unprofessional behaviour. The authors advocate for a more comprehensive and multisource evaluation system in order to identify RID [4].

**M. Lacasse et al. Expectations of Clinical Teachers and Faculty Regarding Development of the CanMEDS-Family Medicine Competencies: Laval Developmental Benchmarks Scale for Family Medicine Residency Training. 2014.**

Uses a modified Delphi methodology to describe the development of expected timeframes for achievement of the CanMEDS Family Medicine (FM) competencies during FM residency training. The authors develop the Laval Developmental Benchmarks Scale for FM, which helps to identify outlier residents who are either excelling or needing remediation [38].

## Discussion

Transitioning to a CBME system requires many policy and practice changes, both at the level of the residency program and in the larger institution. Our research group employed an evidence-informed approach to determining the best practices around resident remediation, and the management of residents in difficulty, by reviewing the literature in these topic areas. Since a more purposeful move toward CBME implementation only occurred very recently, we focused our review on the last 7 years of published literature.

Identifying residents in difficulty, mainly through early and accurate assessment and faculty development, is thought to be a necessary first step in managing these residents. Management requires clear performance benchmarks by competence area, clear definitions and classifications of commonly encountered resident problems, and clear guidelines for identifying specific issues (e.g. how to identify a professionalism issue; how to gauge its severity; determining in which context(s) it is observed). Programs, and their faculty, must understand these criteria so they can to make sure residents in difficulty will be identified early and get access to remediation and other supports as early as possible. Assessments methods (e.g. advocating for more frequent formal and informal evaluation and feedback) and tools need to be improved for better tracking of each resident’s progress during remediation.

There is benefit in tailoring remediation programs to both the specialty and the resident; however, the impacts of these efforts on faculty, health care teams, and patient care must be considered closely. Customization of remediation plans can be very resource intensive. Thus, programs need to determine the appropriate amount of resources to devote to any one resident by considering the type of problem, its severity, and any pertinent resident characteristics (e.g. resident’s personality, which may be more “hard-wired” and thus more difficult to remediate).

Some researchers have pointed to the refinement of postgraduate resident selection processes as a means of limiting the percentage of residents in their programs at risk of requiring remediation. Factors such as past medical school performance can predict learner difficulty later in postgraduate training. Thus, it may be useful to consider such factors when screening applicants for entry to residency programs.

When interventions were mentioned, key information and uniformity were lacking across studies. The study by Meinema et al. encourages other authors to use a checklist to enable more “complete, comparable, and replicable descriptions of educational interventions” (2019) [39].

Programs will need to do some thinking around how the results of this review can best be applied to their program, for the following reasons:

### Most findings are not specific to CBME systems

Despite the increasing popularity of CBME systems globally, very few articles explicitly discussed CBME in the context of resident remediation and/or residents in difficulty. In the last 2 years, the number of articles discussing CBME increased from 14 to 30%. A key implication of these findings is that universities and programs will need to translate the research findings around resident remediation to make them applicable and/or functional for their specific CBME frameworks.

Systems to oversee the promotion of residents from year to year or phase to phase were also rarely discussed. This is worrying. As more and more programs aim to implement CBME, designing better systems for oversight of resident promotion will become a crucial element in ensuring each resident’s competence throughout each stage of training.

### Many of the articles may not be based in the same geographic context or may be specific to a particular specialty

As most studies were either conducted in the United States and/or published by American authors, it will be important for readers to consider the generalizability of these results. For example, attention must be paid to the different educational context(s) in which they were applied.

### The literature focuses on identifying, characterizing, assessing, and remediating only certain types of resident problems

In about a quarter of the articles, remediation was not discussed relative to, or in association with, a particular specialty. Programs will have to consider whether the solutions offered in such a way can be applied to their particular specialty. Nonetheless, even if one’s specialty is not captured in this review, it might be useful to consider the practices of others so as to encourage innovative alternatives. The reviewed literature focused predominantly on the three competency areas of Medical Expert, Professionalism, and Communicator. This may be particularly useful if these are the sorts of resident problem areas that need to be managed locally.

While the findings are largely based in traditional, time-based models of education, this review can still offer general principles to guide the implementation of CBME systems for managing residents in difficulty.

#### Findings of CBME sub-analysis

From the CBME sub-analysis there continues to be an emphasis on identifying RID with more novel assessment methods, which are linked to milestones. Additionally, many articles describe increased frequency of assessment and link that to the role of either an Early Identification Competency Committee (Walburton [33]) or CCC (Domen [24], Ketteler [36], Kinnear [32]). These studies typically describe the members of the CCC and the frequency of meetings, along with some general statements regarding the identification of RID and tracking of progress.

A core premise with CBME is that learners will take different lengths of time to achieve competency in certain milestones. Knowing when a learner is “falling behind” versus on a slower trajectory is one of the difficult components in identifying RID. Lacasse et al. [38] developed the Laval Developmental Benchmark Scale (LDBS) for Family Medicine CanMEDS competencies using a Delphi methodology. They identified RID by establishing outliers based on expected developmental timelines. This work highlights the need for further research into identifying at what point on a continuum learners need more coaching (“little R” remediation) versus more formal remediation within the context of CBME.

The increase in frequency of CBME related articles in the 2015–2017 time period is encouraging. The domains from Van Melle’s framework most often map to “outcome competencies” and “programmatic assessment.” [1] However, there is a paucity of literature on progressive sequencing, and how instructional methods and learning experiences are tailored to those trainees.

#### Limitations and strengths

It is possible that the authors missed papers in their search and follow up strategies.

A limitation of the scoping review methodology, used in this paper, is that the appraisal process does not have the same rigour as a systematic review. This should be kept in mind when interpreting the results. Scoping reviews offer a bird’s-eye view of a topic and can identify key themes. This study is best used to inform about general trends and stimulate more in-depth research in the topic areas discussed.

Additionally, a small number of studies (*n* = 11) were found that specifically studied residents in difficulty who were in a competency-based curriculum. More research reports on residents in competency-based curricula with identified performance problems would help inform the many educators now using competency-based approaches.

A strength of this paper is the experience among the research team in both the topic and methodology: Residents in Difficulty (JP, SGT) and Scoping Reviews (LSA, SGT). Given that the use of competency-based residency educational approaches is accelerating, this review can serve as a baseline for what is known at or near the beginning, with future studies and more in-depth studies building on this starting point.

#### Suggestions for further research

More longitudinal studies on the impact of both competency-based approaches and remediation are needed. There is also a need to understand the effectiveness of different approaches to supporting the success of residents in difficulty. Some of the arguments about the value of competency-based approaches is that underperformance will be uncovered earlier, with more specificity of problems, with additional assessments. Research to explore the accuracy of these assertions would be helpful.

While the findings of this review are largely based in traditional, time-based models of education, it can still offer general principles that can guide the implementation of CBME systems for managing residents in difficulty.

## Conclusion

The major themes identified in both the non-CBME and CBME articles were “identification of deficiencies” and “importance of defining and classifying resident problems or deficiencies as a first step to remediating them.” There were twice as many articles related to CBME in the last 2 years of the review as in the previous 5 years. Several studies described increased attention to identifying RID using milestones and the role of the competence committees.

Mapping of major themes to Van Melle’s [1] framework for CBME programs showed that much work still needs to be done to describe sequenced progression, tailored learning experiences, and competency-focused instruction. Further, there is little literature on the outcomes of remediation in CBME programs. Finally, more studies are needed that specifically describe the processes or interventions in sufficient detail to make them applicable and/or functional for the CBME frameworks of other Universities and programs.

## Supplementary information

**Additional file 1:Supplement A:** References of Articles Reviewed.

**Additional file 2: Supplement B:** Sample Search Strategy.

**Additional file 3: Supplement C:** List of Abstracting Criteria and their Descriptions.

**Additional file 4: Supplement D:** Annotated Bibliography.

**Additional file 5: Supplement E:** Other Important Resources.

**Additional file 6: Supplement F:** Summary of Remediation Practices from the Literature.

## Data Availability

All data generated or analysed during this study are included in this published article [and its supplementary information files].

## Abbreviations

ACGME: Accreditation Council for Graduate Medical Education
BOE: Board of Examiners
BPEA: Best Practices in Evaluation and Assessment
CanMEDS: Canadian Medical Education Directives for Specialists
CBE: Competency Based Education
CBME: Competency Based Medical Education
CCC: Clinical Competency Committee
CD: Critical Deficiencies
DFCM: Department of Family and Community Medicine
DIO: Designated Institutional Offices
EIRC: Early Intervention Remediation Committee
EMBASE: Excerpta Medica dataBASE
ERIC: Education Resources Information Center
FM: Family Medicine
GME: Graduate Medical Education
IM: Internal Medicine
JP: Jonathan Pirie
LDBS: Laval Developmental Benchmark Scale
LSA: Lisa St. Amant
MEDLINE: Medical Literature Analysis and Retrieval System Online
MR: Mariela Ruétalo
OSCE: Objective Standardized Clinical Examinations
PD: Program Director
PG: Postgraduate
RID: Residents in Difficulty
SDOT: Standardized Direct Observation Tools
SGT: Susan Glover Takahashi

## Footnotes

1 Remediation refers to the identification, intervention, and assessment processes at the individual and systems levels.

2 MR - Mariela Ruétalo. Research Officer, Post MD Education, University of Toronto

3 The Best Practices in Evaluation and Assessment (BPEA) Working Group was established 2016 and worked for about 18 months to help the University of Toronto Postgraduate Medical Education Office inform best practices in the area of resident evaluation and assessment to support the curricular redesign to include a competency-based approach. Information was gathered in nine areas including residents in difficulty, remediation, and board of examiners. The BPEA Working Group including its 9 teams reviewed the literature, conducted focus groups, and consulted experts, leading to the identification of recommended priority actions in the area of resident evaluation and assessment for competency based educational programs.
