# Supplementary material for: Managing residents in difficulty within CBME residency educational systems: a scoping review
Source: BMC Med Educ. 2020 Jul 23;20:235. doi: 10.1186/s12909-020-02150-0 (PMC7376876; doi:10.1186/s12909-020-02150-0)
Supplement: Supplementary file 1 — Additional file 1:Supplement A: References of Articles Reviewed. [file 12909_2020_2150_MOESM1_ESM.docx]

## Supplement A. References of Articles Reviewed

1. Aldeen AZ, Salzman DH, Gisondi MA, Courtney DM. Faculty prediction of in-training examination scores of emergency medicine residents. The Journal of emergency medicine. 2014;46(3):390-395.
2. Anderson F, Cachia PG, Monie R, Connacher AA. Supporting trainees in difficulty: a new approach for Scotland. Scottish medical journal. 2011;56(2):72-75.
3. Aram N, Brazil V, Davin L, Greenslade J. Intern underperformance is detected more frequently in emergency medicine rotations. Emergency medicine Australasia: EMA. 2013;25(1):68-74.
4. Audetat M-C, Dory V, Nendaz M, et al. What is so difficult about managing clinical reasoning difficulties? Medical education. 2012;46(2):216-227.
5. Audetat M-C, Laurin S, Dory V. Remediation for struggling learners: putting an end to 'more of the same'. Medical education. 2013;47(3):230-231.
6. Audétat MC, Voirol C, Béland N, Fernandez N, Sanche G. Remediation plans in family medicine residency [Projet de mesures correctives pour les résidents en médecine familiale]. Can Fam Physician. 2015;61(9):e425–e434.
7. Balkany T, Lo K, Francis H, et al. Development and Validation of the Cochlear Implant Surgical Competency Assessment Instrument. Otology & neurotology: official publication of the American Otological Society, American Neurotology Society [and] European Academy of Otology and Neurotology. 2017;38(4):504-509.
8. Barnhoorn PC. Resident remediation: Start from scratch: To the editor. *American Journal of Clinical Pathology.* 2015;144(3):525.
9. Barnhoorn PC, Domen RE. Resident remediation: Start from scratch: The author's reply. *American Journal of Clinical Pathology.* 2015;144(3):525-526.
10. Bhatti NI, Ahmed A, Stewart MG, Miller RH, Choi SS. Remediation of problematic residents - A national survey. Laryngoscope. 2016;126(4):834-838.
11. Casaletto JJW, Michael C.; Ankel, Felix K.; Bourne, Christina L. and Ghaemmaghami, Chris A. Emergency Medicine Rural Rotations: A Program Director's Guide. Annals of Emergency Medicine. 2013;61(5).
12. Cleland J, Leggett H, Sandars J, Costa MJ, Patel R, Moffat M. The remediation challenge: theoretical and methodological insights from a systematic review. Medical education. 2013;47(3):242-251.
13. Dalack GW, Jibson MD. Clinical skills verification, formative feedback, and psychiatry residency trainees. Academic psychiatry: the journal of the American Association of Directors of Psychiatric Residency Training and the Association for Academic Psychiatry. 2012;36(2):122-125.
14. de Montbrun S, Louridas M, Grantcharov T. Passing a Technical Skills Examination in the First Year of Surgical Residency Can Predict Future Performance. Journal of graduate medical education. 2017;9(3):324-329.
15. Derderian CA, Kenkel JM. Remediation as a Road to Competency: Strategies for Early Identification of the Struggling Resident and Generating the Remediation Plan. Journal of Craniofacial Surgery. 2016;27(1):8-12.
16. Domen RE. Resident remediation, probation, and dismissal basic considerations for program directors. American journal of clinical pathology. 2014;141(6):784-790.
17. Domen RE, Johnson K, Conran RM, et al. Professionalism in Pathology: A Case-Based Approach as a Potential Educational Tool. Archives of pathology & laboratory medicine. 2017;141(2):215-219.
18. Dupras DM, Edson RS, Halvorsen AJ, Hopkins RH, Jr., McDonald FS. "Problem residents": prevalence, problems and remediation in the era of core competencies. The American journal of medicine. 2012;125(4):421-425.
19. Gas BL, Buckarma EH, Mohan M, Pandian TK, Farley DR. Objective Assessment of General Surgery Residents Followed by Remediation. Journal of Surgical Education. 2016;73(6):e71-e76.
20. Gaviola G, Smith SE. Assessment of fellowship trainee clinical competency and growth with an objective standardized clinical examination within the musculoskeletal fellowship program: Initial experience. 2012 Annual Meeting of the Society of Skeletal Radiology; 2012; Miami Beach, FL, USA.
21. Guerrasio J, Aagaard EM. Methods and outcomes for the remediation of clinical reasoning. Journal of general internal medicine. 2014;29(12):1607-1614.
22. Guerrasio J, Brooks E, Rumack CM, Christensen A, Aagaard EM. Association of Characteristics, Deficits, and Outcomes of Residents Placed on Probation at One Institution, 2002-2012. Academic Medicine. 2016;91(3):382-387.
23. Guerrasio J, Garrity MJ, Aagaard EM. Learner deficits and academic outcomes of medical students, residents, fellows, and attending physicians referred to a remediation program, 2006-2012. Academic medicine: journal of the Association of American Medical Colleges. 2014;89(2):352-358.
24. Guevara M, Grewald Y, Hutchinson K, Amoateng-Adjepong Y, Manthous C. Individualized education plans in medical education. Connecticut medicine. 2011;75(9):537-540.
25. Jones AT, Biester TW, Buyske J, Lewis FR, Malangoni MA. Using the American Board of Surgery In-Training Examination to predict board certification: a cautionary study. Journal of surgical education. 2014;71(6):e144-148.
26. Katz ED, Goyal DG, Char D, Coopersmith CM, Fried ED. A novel concept in residency education: case-based remediation. The Journal of emergency medicine. 2013;44(2):493-498.
27. Kedian T, Gussak L, Savageau JA, et al. An ounce of prevention: how are we managing the early assessment of residents' clinical skills? A CERA study. Family medicine. 2012;44(10):723-726.
28. Ketteler ER, Auyang ED, Beard KE, et al. Competency champions in the clinical competency committee: a successful strategy to implement milestone evaluations and competency coaching. Journal of surgical education. 2014;71(1):36-38.
29. Kinnear B, Bensman R, Held J, O'Toole J, Schauer D, Warm E. Critical Deficiency Ratings in Milestone Assessment: A Review and Case Study. Academic medicine: journal of the Association of American Medical Colleges. 2017;92(6):820-826.
30. Krzyzaniak SM, Wolf SJ, Byyny R, et al. A qualitative study of medical educators' perspectives on remediation: Adopting a holistic approach to struggling residents. Medical teacher. 2017;39(9):967-974.
31. Lacasse M, Theoret J, Tessier S, Arsenault L. Expectations of clinical teachers and faculty regarding development of the CanMEDS-Family Medicine competencies: Laval developmental benchmarks scale for family medicine residency training. Teaching and learning in medicine. 2014;26(3):244-251.
32. Leung F-H, Ratnapalan S. A framework to teach self-reflection for the remedial resident. Medical teacher. 2011;33(3):e154-157.
33. Magin P, Stewart R, Turnock A, Tapley A, Holliday E, Cooling N. Early predictors of need for remediation in the Australian general practice training program: a retrospective cohort study. Advances in health sciences education: theory and practice. 2017;22(4):915-929.
34. Mar C, Chang S, Forster B. Remedial training for the radiology resident: a template for optimization of the learning plan. Academic radiology. 2015;22(2):240-246.
35. McMurray L, Hall AK, Rich J, Merchant S, Chaplin T. The Nightmares Course: A Longitudinal, Multidisciplinary, Simulation-Based Curriculum to Train and Assess Resident Competence in Resuscitation. Journal of graduate medical education. 2017;9(4):503-508.
36. O'Neill LD, Norberg K, Thomsen M, et al. Residents in difficulty--just slower learners? A case-control study. BMC medical education. 2014;14:1047.
37. Papadakis MA, Paauw DS, Hafferty FW, Shapiro J, Byyny RL, Alpha Omega Alpha Honor Medical Society Think T. Perspective: the education community must develop best practices informed by evidence-based research to remediate lapses of professionalism. Academic medicine: journal of the Association of American Medical Colleges. 2012;87(12):1694-1698.
38. Platt MP, Davis EM, Grundfast K, Grillone G. Early detection of factual knowledge deficiency and remediation in otolaryngology residency education. The Laryngoscope. 2014;124(8):E309-311.
39. Puscas L. Otolaryngology resident in-service examination scores predict passage of the written board examination. Otolaryngology--head and neck surgery: official journal of American Academy of Otolaryngology-Head and Neck Surgery. 2012;147(2):256-260.
40. Riebschleger MP, Haftel HM. Remediation in pediatric residency programs: A survey of pediatric program directors. *Academic Paediatrics.* 2011;11(4):e7.
41. Roberts NK, Williams RG, Klingensmith M, et al. The case of the entitled resident: a composite case study of a resident performance problem syndrome with interdisciplinary commentary. Medical teacher. 2012;34(12):1024-1032.
42. Roy BC, Shobhina G.; Bates, Carol; Dunn, Kathel; Karani, Reena and Willett, Lisa L. . For the General Internist: A Summary of Key Innovations in Medical Education. J Gen Intern Med 2016;31(8):941-946.
43. Rumack CM, Guerrasio J, Christensen A, Aagaard EM. Academic Remediation: Why Early Identification and Intervention Matters. Academic radiology. 2017;24(6):730-733.
44. Saloum D, Bialeck S, Motov S, Rose J, Aghera A. An effective remediation program to improve in-training exam scores. *Academic Emergency Medicine.* 2013;20(5 Suppl. 1):S56.
45. Sanfey H, Darosa DA, Hickson GB, et al. Pursuing professional accountability: an evidence-based approach to addressing residents with behavioral problems. Archives of surgery (Chicago, Ill : 1960). 2012;147(7):642-647.
46. Santen S, Taira T, Roberts N. How do we remediate the problem resident? *Annals of Emergency Medicine* 2012;60(5):S167.
47. Sanfey H, Williams R, Dunnington G. Recognizing residents with a deficiency in operative performance as a step closer to effective remediation. Journal of the American College of Surgeons. 2013;216(1):114-122.
48. Schenarts PJ, Langenfeld S. The Fundamentals of Resident Dismissal. The American surgeon. 2017;83(2):119-126.
49. Schwed AC, Lee SL, Salcedo ES, et al. Association of General Surgery Resident Remediation and Program Director Attitudes with Resident Attrition. JAMA surgery. 2017;152(12):1134-1140.
50. Sharma R, Sperling JD, Greenwald PW, Carter WA. A novel comprehensive in-training examination course can improve residency-wide scores. Journal of graduate medical education. 2012;4(3):378-380.
51. Smith JL, Lypson M, Silverberg M, et al. Defining Uniform Processes for Remediation, Probation and Termination in Residency Training. The western journal of emergency medicine. 2017;18(1):110-113.
52. Sparks JW, Landrigan-Ossar M, Vinson A, et al. Individualized remediation during fellowship training. Journal of Clinical Anesthesia. 2016;34:452-458.
53. Stirling K, Hogg G, Ker J, Anderson F, Hanslip J, Byrne D. Using simulation to support doctors in difficulty. The clinical teacher. 2012;9(5):285-289.
54. Taggarshe D, Mittal V. The utility of the ABS in-training examination (ABSITE) score forms: percent correct and percentile score in the assessment of surgical residents. Journal of surgical education. 2012;69(4):554-558.
55. Taira T, Roberts N, Santen S. Defining professionalism issues among "problem residents". *Annals of Emergency Medicine.* 2012;60(5):S167.
56. Taira T, Santen S, Roberts N. Developing a taxonomy of "problem residents". *Annals of Emergency Medicine.* 2012;60(5):S169.
57. Turner JA, Fitzsimons MG, Pardo MC, et al. Effect of Performance Deficiencies on Graduation and Board Certification Rates: A 10-yr Multicenter Study of Anesthesiology Residents. Anesthesiology. 2016;125(1):221-229.
58. Visconti A, Bhat R, Levine B, Garg M, Takenaka K. Predictors of chief resident appointment or the need for remediation of emergency medicine residents. Academic Emergency Medicine. 2016;23:S257.
59. Warburton KM, Goren E, Dine CJ. Comprehensive Assessment of Struggling Learners Referred to a Graduate Medical Education Remediation Program. Journal of graduate medical education. 2017;9(6):763-767.
60. Webb TP, Paul J, Treat R, Codner P, Anderson R, Redlich P. Surgery residency curriculum examination scores predict future American Board of Surgery in-training examination performance. Journal of surgical education. 2014;71(5):743-747.
61. Weizberg M, Smith JL, Murano T, Silverberg M, Santen SA. What does remediation and probation status mean? A survey of emergency medicine residency program directors. Academic emergency medicine: official journal of the Society for Academic Emergency Medicine. 2015;22(1):113-116.
62. Yost MJ, Gardner J, Bell RM, et al. Predicting academic performance in surgical training. Journal of surgical education. 2015;72(3):491-499.
63. Zbieranowski I, Takahashi SG, Verma S, Spadafora SM. Remediation of residents in difficulty: a retrospective 10-year review of the experience of a postgraduate board of examiners. Academic medicine: journal of the Association of American Medical Colleges. 2013;88(1):111-116.
