# Supplementary material for: Managing residents in difficulty within CBME residency educational systems: a scoping review
Source: BMC Med Educ. 2020 Jul 23;20:235. doi: 10.1186/s12909-020-02150-0 (PMC7376876; doi:10.1186/s12909-020-02150-0)
Supplement: Supplementary file 2 — Additional file 2: Supplement B: Sample Search Strategy. [file 12909_2020_2150_MOESM2_ESM.docx]

## Supplement B: Sample Search Strategy

RID Literature Review Search Strategy & Results – 2011 to Oct 11, 2018

Inclusion Criteria:

1. Must be about postgraduate medical education
2. Must be about residents in difficulty
3. Must offer information to inform structure (guidelines; program, design; includes promotion and progress) and processes (features of competence, e.g. CanMEDS Roles involved) of competence

MEDLINE – Search Strategy

1. trainee.mp.
2. intern.mp.
3. residency.mp.
4. house officer.mp.
5. education, medical, graduate/
6. exp Remedial Teaching/
7. remediation.mp.
8. academic difficulty.mp.
9. residents in difficulty.mp.
10. resident in difficulty.mp.
11. 1 or 2 or 3 or 4 or 5
12. 6 or 7 or 8 or 9 or 10
13. 11 and 12
14. Limit to (English language and yr=2011 – current)
