# Supplementary material for: Managing residents in difficulty within CBME residency educational systems: a scoping review
Source: BMC Med Educ. 2020 Jul 23;20:235. doi: 10.1186/s12909-020-02150-0 (PMC7376876; doi:10.1186/s12909-020-02150-0)
Supplement: Supplementary file 3 — Additional file 3: Supplement C: List of Abstracting Criteria and their Descriptions. [file 12909_2020_2150_MOESM3_ESM.docx]

## Supplement C: List of Abstracting Criteria and their Descriptions

**BPEA Literature Review on Residents in Difficulty, Remediation, BOE**

**Abstracting Criteria Definitions**

| **Item #** | **Abstracting Criteria** | | **Description** | **Examples** |
| --- | --- | --- | --- | --- |
|  | Reviewer initials | | Four reviewers were involved in the secondary screening of articles: JP, LSA, MR, and SGT. |  |
|  | Last name of first author | | | |
|  | Publication year | | | |
|  | ISSN or DOI number (if applicable/available) | | | |
|  | Type of article | | Article type includes commentaries, editorials, letters, history articles, review articles, primary research articles, and “other” (e.g. conference publications). |  |
|  | After reviewing the full text, does the article still meet our previous eligibility criteria? | | Articles are deemed eligible if they:  (1) are about postgraduate medical education;  (2) are about residents in difficulty;  (3) offer information to inform structure and/or processes of competence.  If articles are not eligible OR the reviewer is “unsure,” the reviewer is to describe their reasoning for either decision. |  |
|  | Location of author(s)  (check all that apply) | | Includes the following locations: USA, Canada, Europe, Australia and New Zealand, and “other” (for those not included in this list). |  |
|  | Location of study  (check all that apply) | | Includes the following locations: USA, Canada, Europe, Australia and New Zealand, and “other” (for those not included in this list). |  |
|  | Learner PGY level(s)  (check all that apply) | | Includes PGY1 to PGY5 and “other” levels of postgraduate education, such as a house officer (U.K.). |  |
|  | Learner residency program / specialty | | Select all programs/ specialties discussed in the article. “Other” to indicate any other programs/ specialties outside those listed in the abstracting form. |  |
|  | What area(s) of competence were discussed? (check all that apply) | | Includes discussions of competence as it pertains to the Medical Expert, Communicator, Collaborator, Leader, Health Advocate, Scholar, and Professional CanMEDS 2015 Roles. There is also an “other” category for discussions of competence outside the CanMEDS Roles. |  |
|  | What “structures to support competence” were discussed, as it relates to residents in difficulty, remediation and/or BOE?  (check all that apply) | Guidelines for resident | Guiding principles, suggestions, and/or lessons for residents; what a resident should be doing to help support / develop / improve their competence. | Effective study habits / strategies; time management |
|  |  | Guidelines for program | Guiding principles, suggestions, and/or lessons to help support resident competence. | Planned meetings with residents to assess progress; suggestions to help programs identify, monitor, and/or remediate residents in difficulty |
|  |  | Guidelines for hospital / health facility | Guiding principles, suggestions, and/or lessons for hospitals / health facilities to help support resident competence. | Around hospital orientation materials – i.e. preparing residents for residency; includes discussions of international residency programs that are hospital-based and not accountable to the university (unlike many programs in Canada) |
|  |  | Guidelines for university | Guiding principles, suggestions, and/or lessons for the university (e.g. Postgraduate Medical Education and BOE) that help to support the competence of residents and the successful remediation of those in difficulty. |  |
|  |  | Design of individual resident educational plan/program | Includes discussion on measures of competence, types of assessment tools, educational interventions, timelines. |  |
|  |  | Design of residency educational program | Describes the features of an educational program as it relates to residents in difficulty, remediation, and/or BOE. | Program goals, assessment tools, coaches |
|  |  | Assessment tool(s) | Articles that specifically discuss (an) assessment tool(s) (e.g. tool type – MSF, mini-CEX, ITER, etc. – items/content, goals and objectives, validity, effectiveness) in terms of either identifying / predicting residents in difficulty or evaluating residents in remediation. |  |
|  |  | Promotion of resident systems (i.e. from one PG year to another) | Discussion of how residents are promoted (e.g. what assessment criteria, type, and quantity are considered; what determines pass/fail) and how this supports competence. |  |
|  |  | Other |  |  |
|  | In follow-up to question 12, please provide further detail on the structure(s) to support competence mentioned in this article and discuss its/ their relative value to our research question. | | How are these structures to support competence discussed? What are the implications? How do the structures discussed inform best practices in evaluation and assessment as they relate to residents in difficulty, remediation, and/or BOE? |  |
|  | Was a competency-based educational orientation or framework used in this article (as defined and applied by the author(s))? | | Did the author(s) use / apply a competency-based educational orientation / framework in the article?  Note that the “competency-based educational orientation / framework” is as per described / defined by the author(s). |  |
|  | Please briefly state the purpose of the article. | | | |
|  | Please provide any additional observations or comments related to residents in difficulty, remediation, and/or BOE. | | | |
