# Supplementary material for: Managing residents in difficulty within CBME residency educational systems: a scoping review
Source: BMC Med Educ. 2020 Jul 23;20:235. doi: 10.1186/s12909-020-02150-0 (PMC7376876; doi:10.1186/s12909-020-02150-0)
Supplement: Supplementary file 4 — Additional file 4: Supplement D: Annotated Bibliography. [file 12909_2020_2150_MOESM4_ESM.docx]

## Supplement D: Annotated Bibliography

**Audétat, M.-C., C. Voirol, N. Béland, N. Fernandez and G. Sanche. (2015). "Remediation plans in family medicine residency." *Canadian Family Physician* 61(9): e425-e434.**

Audétat et al. developed an instrument to evaluate remediation plans from different teaching sites. A total of 23 plans were analysed across 10 teaching sites. The instrument was used to assess the content, process, and quality of remediation plans, as well as students’ academic and rotation assessment results before and after their participation in the remediation plan. The authors identified criteria for good remediation plans, which included diagnosing and describing the deficiency, stating the objectives and duration of remediation, and having someone oversee and be responsible for the remediation process.^6^

Key theme: remediation plan criteria

**Domen, R. E. (2014). "Resident remediation, probation, and dismissal: basic considerations for program directors." *American Journal of Clinical Pathology* 141(6): 784-790.** ^8^

This paper offers an 8-step approach to the development of a remediation or probation plan. For example, this article describes how faculty development on the assessment of core competencies and milestones is key to residents’ success and can help ensure that resident deficiencies are identified early. Identifying resident deficiencies early is also facilitated through timely and truthful evaluations with effective feedback on performance. Other recommendations were that the remediation plan should only target the specific issue(s) identified for remediation; that the plan should have clearly defined goals with defined measures/assessments; and that there need to be realistic timelines for achievement, designated faculty/mentors to assist the resident, and regular meeting dates to assess progress.

Key themes: competency framework, competence committee, remediation/probation plan, deficiency identification

**Ketteler, E. R., E. D. Auyang, K. E. Beard, E. L.McBride, R. McKee, J. C. Russell, N. L.Szoka and M. T. Nelson. (2014). "Competency Champions in the Clinical Competency Committee." *Journal of Surgical Education* 71(1): 36-38.**^29^

This article describes how a clinical competence committee (CCC), using the ACGME competency-based framework, was developed to guide remediation and the coaching of residents in difficulty.

Faculty “champions” were appointed by the Program Director and assigned ACGME competencies, based on their expertise in each area. The “champions” were found to be effective in coaching and in providing clear tangible objectives for residents to aim towards, providing indications of success or being “back on track.”

There were a total of 12 CCC members and meetings were held monthly. Having monthly meetings was said to prompt early discussion of residents in difficulty. The authors found that most residents identified to be in difficulty really required coaching (informal) rather than remediation (formal). Formal remediation was thus left for residents who did not progress with coaching.

The authors also found that faculty improved in their ability to assess residents and define the objectives centred on required competencies. They also became skilled in providing structured feedback *throughout* the rotation, rather than just at the end.

While *initially* time-consuming, faculty on the CCC learned, within the span of a year, how to review an entire class of residents in an hour. As a result of the CCC, expectations of residents became clearer and the milestones more observable and tangible. The CCC not only helped to better assess residents, but also improved rotations to ensure that they provided the clinical experiences necessary to help residents achieve all competencies.

Key theme: competency framework

**Lacasse, M., J. Théorêt, S. Tessier and L. Arsenault. (2014). "Expectations of clinical teachers and faculty regarding development of the CanMEDS-Family Medicine competencies: Laval developmental benchmarks scale for family medicine residency training." *Teaching and Learning in Medicine* 26(3): 244-251.^41^**

The authors of this article set out to map CanMEDS-FM competencies to different developmental stages of training in Family Medicine (FM), by consulting experts using Delphi methodology. They found that most benchmarks under the Medical Expert, Collaborator, and Health Advocate Roles should be achieved in between 6 and 18 months of training. Competencies under the Manager Role, and most of those pertaining to the Scholar Role, should be achieved by the second year of residency or into their early practice. Competencies under the Professional and Communicator Roles should be achieved early in practice. This benchmark scale can be useful for teaching and evaluating residents, as well as for enabling better/earlier identification of residents in difficulty.

Key theme: competency framework

This report also offers helpful information on investigating and tackling resident difficulties, and on assigning and defining roles and responsibilities in remediating issues, among other things. The authors claim that systems should be aimed toward recognizing early warning signs of resident problems in order to intervene before the problem has repercussions (e.g. to patient safety) and the problem becomes “hardwired,” rather than focusing energies on “crisis management.” Three categories of problems are listed and described (personal conduct, professional conduct, and professional competence).

Key themes: deficiency definitions and/or classification systems, deficiency identification
