# Supplementary material for: Managing residents in difficulty within CBME residency educational systems: a scoping review
Source: BMC Med Educ. 2020 Jul 23;20:235. doi: 10.1186/s12909-020-02150-0 (PMC7376876; doi:10.1186/s12909-020-02150-0)
Supplement: Supplementary file 5 — Additional file 5: Supplement E: Other Important Resources. [file 12909_2020_2150_MOESM5_ESM.docx]

## Supplement E: Other Important Resources

**Audétat, M.-C., V. Dory, M. Nendaz, D. Vanpee, D. Pestiaux, N. Junod Perron and B. Charlin. (2012). "What is so difficult about managing clinical reasoning difficulties?" *Medical Education* 46(2): 216-227**.^4^

Audétat et al. describe a model for faculty development that can help to improve early and accurate diagnoses of residents’ deficiencies in the area of clinical reasoning.

Key themes: faculty development and support

**Audétat, M.-C., S. Laurin and V. Dory. (2013). "Remediation for struggling learners: putting an end to 'more of the same'." *Medical education* 47(3): 224-231.^5^**

Audétat et al. describe a four-pronged approach to supporting clinical teachers in remediating residents. For example, they discuss faculty development efforts to introduce clinical teachers to conceptual frameworks and empirical findings around remediation strategies, as well as providing the teachers with training to develop their teaching and assessment skills.

Key themes: faculty development and support

**Anderson, F., P. G. Cachia, R. Monie and A. A. Connacher. (2011). "Supporting trainees in difficulty: a new approach for Scotland." *Scottish Medical Journal* 56(2): 72-75**.^23^

Using their operational framework, Anderson et al. offer practical guidance to residency training programs for supporting residents in difficulty.

Key theme: operational framework

**Cleland, J. A., H. Leggett, J. Sandars, M. J. Costa, R. Patel and M. Moffat. (2013). "The remediation challenge: theoretical and methodological insights from a systematic review." *Medical Education* 47(3): 242-251.^1^**

In the most recent literature review on remediation, conducted by Cleland et al., the quality of literature was found to be quite low (e.g. no controls, small sample sizes) making it difficult to delineate the most effective remediation methods. In addition, most remediation methods equate success with passing an examination or assessment(s). They do not offer further insight as to how much additional work or teaching is needed to develop residents’ learning. More recently published articles were observed to be of higher quality.

Key theme: literature review on remediation

**Dupras, D. M., R. S. Edson, A. J. Halvorsen, R. H. Hopkins, Jr. and F. S. McDonald. (2012). "Problem residents": prevalence, problems and remediation in the era of core competencies." *American Journal of Medicine* 125(4): 421-425.^40^**

Using the Accreditation Council for Graduate Medical Education (ACGME) framework, this article discusses the prevalence of “problem residents,” the contexts in which they were identified, and the methods used by Program Directors to resolve them. The authors found that the majority of residents who were in difficulty had deficiencies in multiple competencies, requiring a comprehensive and multisource evaluation system.

Key theme: competency framework

**Ghaemmaghami, C. A., A. Sudhir and W. A. Woods. (2012). "The incorporation of high-fidelity simulation in the evaluation of efficacy of a residency remediation plan." *Academic Emergency Medicine* 19(4 (Supplement 1)): S400.^28^**

This article describes a successful program of assessment for all of the ACGME competencies (e.g. through the use of written essay examinations, oral board-type examinations, and high-fidelity simulations). Oral board and simulation cases were evaluated using case-based critical action checklists and a Standardized Direct Observation Tool (SDOT). The authors argue that subjective measures are often used to assess the efficacy of a remediation program and that this is problematic.

Key theme: competency framework

**Guerrasio, J. and E. M. Aagaard. (2014). "Methods and outcomes for the remediation of clinical reasoning." *Journal of General Internal Medicine* 29(12): 1607-1614.**^37^

Guerrasio and Aagaard assessed the effectiveness of a ten-step remediation program targeted toward clinical reasoning deficits. Their program, designed to be 4 to 6 weeks long, was found to have resident outcomes that were comparable to other programs that lasted 6 months. The shorter program duration was deemed beneficial, as this minimized the exposure of underperforming residents to patients, without compromising their performance outcomes. A caveat regarding this remediation program was that it was still time-consuming for faculty participants.

Key theme: remediation plan

**Hodges, B. D., S. Ginsburg, R. Cruess, S. Cruess, R. Delport, F. Hafferty, M. J. Ho, E. Holmboe, M. Holtman, S. Ohbu, C. Rees, O. ten Cate, Y. Tsugawa, W. Van Mook, V. Wass, T. Wilkinson and W. Wade. (2011). "Assessment of professionalism: recommendations from the Ottawa 2010 conference." *Medical Teacher* 33(5): 354-363.**^46^

Members of the International Ottawa Conference Working Group on the Assessment of Professionalism (IOC-PWG) conducted a critical discourse analysis of identified articles on Professionalism. Their analysis offers a classification of the prominent discourses in the literature on professionalism by scope (individual, interpersonal, societal/ institutional) and epistemology (theory behind discourse, i.e. objectivist/ positivist or subjective/ constructivist). Three discourses on professionalism were identified: (1) Professionalism as an individual characteristic, trait, behaviour or cognitive process; (2) Professionalism as an interpersonal process or effect; and (3) Professionalism as a societal/institutional phenomenon. The authors offer recommendations for the assessment of each.

Key themes: deficiency definitions and/or classification systems

**O'Neill, L. D., K. Norberg, M. Thomsen, R. D. Jensen, S. G. Brondt, P. Charles, L. S. Mortensen and M. K. Christensen. (2014). "Residents in difficulty—just slower learners? A case-control study." *BMC Medical Education* 14: 1047.**^26^

The aim of this study was to determine whether and which medical school performance indicators were predictive of difficulties during residency. This was a case-control study of specialist trainees identified to be in difficulty from one university. The authors discovered that the two variables of “time to complete medical school” and “number of re-examinations” predicted difficulties during residency. Average medical school grades were *not* found to be predictive.

Key themes: resident selection; deficiency identification

**Puscas, L. (2012). "Otolaryngology resident in-service examination scores predict passage of the written board examination." *Otolaryngology – Head & Neck Surgery* 147(2): 256-260.^32^**

This was a historical cohort study that identified significant associations between residents’ Otolaryngology Training Examination (OTE) scores and their first-time American Board of Otolaryngology (ABOto) Written Qualifying Exam (WQE) scores. The OTE can therefore serve as a method for identifying residents at risk of failing their WQE. Those scoring in the bottom quartile for the OTE are at significantly greater risk of failing the WQE.

Key theme(s): deficiency identification

**Roberts, N. K., R. G. Williams, M. Klingensmith, M. Sullivan, M. Boehler, G. Hickson, M. J. Kim, D. L. Klamen, T. Leblang, C. Schwind, K. Titchenal and G. L. Dunnington (2012). "The case of the entitled resident: a composite case study of a resident performance problem syndrome with interdisciplinary commentary." *Medical Teacher* 34(12): 1024-1032.**^43^

In this case study, the authors state the importance of balancing the needs of residents in difficulty with the needs of the health care system. This is because remediation takes a lot of time and energy and can adversely affect the functioning of healthcare professional teams. The authors also advise taking early reports of resident problems seriously, as residents tend to be on their best behaviour early on and faculty are more lenient. Faculty are advised not to tolerate behaviour in residents that they would not tolerate in their colleagues. Lastly, institutional, systemic, and systematic support is needed to address residents in difficulty.

Key theme(s): remediation plan; deficiency identification; health care system

**Sanfey, H., D. A. Darosa, G. B. Hickson, B. Williams, R. Sudan, M. L. Boehler, M. E. Klingensmith, D. Klamen, J. D. Mellinger, J. C. Hebert, K. M. Richard, N. K. Roberts, C. J. Schwind, R. G. Williams, A. K. Sachdeva and G. L. Dunnington. (2012). "Pursuing professional accountability: an evidence-based approach to addressing residents with behavioral problems." *Archives of Surgery* 147(7): 642-647.**^18^

Sanfey et al. offer strategies for improving the resident selection process (in the context of identifying residents with behavioural issues), including the use of multiple mini-interviews, provocative OSCEs, and personality testing.

Key theme: resident selection

**Sanfey, H., R. Williams and G. Dunnington (2013). "Recognizing residents with a deficiency in operative performance as a step closer to effective remediation." *Journal of the American College of Surgeons* 216(1): 114-122. ^19^**

The authors undertook a systematic approach to evaluating operative performance by analysing over 1000 comments from expert raters on the perceived strengths and weaknesses of audio-videotaped resident performances.

The authors sought to categorize and describe common deficiencies in surgery. Five categories of deficiencies were identified: technical skills, forward planning, self-direction, situation awareness, and patient safety/judgment. The authors also offer specific tools and evaluation methods for assessing each category of deficiency in terms of both identification and monitoring of progress, and deficiency-specific methods for remediation (see Table 1 of the article for a helpful summary of these findings). Effective feedback, deliberative practice, and specific performance targets were found to be essential components of operative training and remediation.

To help with the early identification of residents in difficulty, faculty are advised that engaging in regular discussions about residents’ performance is necessary.

Key themes: competency framework, deficiency definitions and/or classification systems, deficiency identification
