# Supplementary material for: Managing residents in difficulty within CBME residency educational systems: a scoping review
Source: BMC Med Educ. 2020 Jul 23;20:235. doi: 10.1186/s12909-020-02150-0 (PMC7376876; doi:10.1186/s12909-020-02150-0)
Supplement: Supplementary file 6 — Additional file 6: Supplement F: Summary of Remediation Practices from the Literature. [file 12909_2020_2150_MOESM6_ESM.docx]

## Supplement F: Summary of Remediation Practices from the Literature

### Methods of Monitoring All ****Residents’**** Performance

- 1. Informal and formal methods for addressing areas of concern in residents
  2. Faculty development for giving feedback
  3. Formal methods for catching deficiencies in all competency domains

### Structural and Design Practices

- 1. Defining remediation terms (institutional)
  2. Creating a classification system for categorizing resident problems
  3. Clarifying whether or not wellness issues are associated with resident performance
  4. Developing a system for classifying the level of seriousness of resident problems to help determine the best course of action, whether coaching, remediation, or probation (e.g. see the classification of levels of seriousness offered by Anderson et al.^23^)
  5. Creating guidelines / assessment measures / protocols for identifying residents in difficulty

*For example:*

- The earlier, the better, for identification of residents in difficulty:
  - Identification requires early, accurate assessment of residents to track their progress.
  - There must be clearly outlined, observable, and tangible objectives for residents to meet in all competencies.
  - Program Directors are advised to take very seriously any suggestions of poor behaviour and performance in residents early in training, as it’s usually during this period that residents put their best foot forward and faculty are more lenient / dismissive of problems in their assessments of residents. For this reason, even “neutral” comments / feedback / assessments from faculty need to be followed up on by Program Directors during this period.
  - Review and consider adopting the 10 guiding principles for managing trainees in difficulty as outlined by Anderson et al.^23^

### Identifying Resident Problem(s)

- 1. Faculty are tasked with observing and evaluating residents’ performance and identifying those learners found to be underperforming, or to be at risk of underperforming. Some faculty development is required to enhance faculty ability to diagnose resident deficiencies

### Define / Describe Resident Problem(s)

- 1. This involves investigation of reports made by faculty, or other health professional staff,^27^ to better classify and define the resident’s problem and its level of seriousness

### Tailor Plan to Individual’s Needs

- 1. Modify the existing remediation plan / approach for customization to the individual resident in difficulty (personality, learning style, etc.) and the identified problem (e.g. clinical reasoning)
  2. For guidance, see Domen’s eight steps for the development of a remediation plan^8^

### Define the Roles and Responsibilities of All Players Involved in Remediation

1. Remediation needs a team approach, including the resident, Program Director, faculty, postgraduate leaders, educational design resources, and coaches, including wellness, medical expert, communication, collaboration, and professionalism resources

### Remediate Resident

1. Create structured, transparent educational programs, with additional supports such as coaching or mentorship
2. Focus on primary problems first and limit remediation to a small number of areas at a time

### Assess Resident’s Progress during Remediation and Determine Next Steps

1. Regular assessment, regular monitoring, and effective feedback are necessary for good remediation outcomes

### Attending to Key Elements of CBME Design in Remedial Program Design Likely Beneficial

1. Establish an outcomes-based competency framework
2. Use a sequenced progression of competence
3. Include tailored learning experiences for the achievement of competencies
4. Tailor teaching to competencies, which for residents includes multiple workplaces
5. Observations should be followed by feedback and coaching
6. Take a purposeful and programmatic approach to assessment
